# EU and UK migration policies impeding mental health justice - a critical review

**DOI:** 10.1192/j.eurpsy.2025.1504

**Published:** 2025-08-26

**Authors:** C. Diefenbach

**Affiliations:** 1 Department of Psychiatry, Psychosomatic Medicine and Psychotherapy, Goethe University Frankfurt, University Hospital, Frankfurt am Main, Germany

## Abstract

**Introduction:**

Ongoing global conflicts have led to increasing forced migration and displacement. This rising numbers’ trend has fueled discriminatory policies in the EU and UK, resulting in detrimental mental health consequences for refugees and asylum seekers (RAS) caused by post-migration stressors. These policies sharply contrast ratified treaties and conventions based on the Universal Declaration of Human Rights (UDHR).

**Objectives:**

The review examines the infringement of RAS’ right to health, focusing on the mental health consequences of post-migration stressors faced upon arrival in the EU and the UK. It analyses how these post-migration stressors reflect a violation of human rights (HR), tracing the evolution of human rights theory from natural law to contemporary universal principles. It investigates how post-migration stressors exacerbate psychiatric symptoms in an already vulnerable population present in the EU and UK. It seeks to understand whether the EU faces a moral cosmopolitan duty of humanitarianism towards RAS, exploring the ethical and legal obligations of the EU and UK under key international HR frameworks.

**Methods:**

A systematic search was conducted across several databases, including PubMed, ScienceDirect, and GoogleScholar. The search comprised keywords such as human rights, cosmopolitanism, health justice, post-migration stressors, refugees and asylum seekers, common european asylum system and mental health. Boolean Operators AND and OR were applied. Exclusion criteria included non-English/German/Italian publications, papers focused on non-european asylum policies or refugee populations outside the EU and UK, and study protocols. Studies were synthesised to provide a comprehensive overview of post-migration stressors in the EU and UK and a philosophical deduction of human rights.

**Results:**

Both the EU and UK violate international HR law by failing to ensure the right to health for RAS. Empirical studies from Greece, Italy, and Germany document that post-migration stressors—such as inadequate housing, poor living conditions, and delays in asylum procedures—exacerbate mental health conditions like depression, post-traumatic-stress disorder, and anxiety. The recent reform of the Common European Asylum System (CEAS) demonstrates the gap between legal commitments in international law and current migration policies. This further restricts access to asylum and (mental) healthcare, subjecting RAS to violence and inadequate care.

**Conclusions:**

The EU’s and UK’s political and legal responses violate basic human rights of RAS, particularly their right to health, despite legal commitments to international HR treaties. This further exacerbates mental health conditions in RAS. A human rights-based approach, integrating mental health into migration policies, is crucial to protecting RAS’ dignity and mental health. Until then, the EU and UK will continue to fall short of their moral and legal obligations.

**Disclosure of Interest:**

None Declared

